# Supplementary material for: The transcriptional landscape of Venezuelan equine encephalitis virus (TC-83) infection
Source: PLoS Negl Trop Dis. 2021 Mar 31;15(3):e0009306. doi: 10.1371/journal.pntd.0009306 (PMC8041203; doi:10.1371/journal.pntd.0009306)
Supplement: S1 Text — (DOCX) [file pntd.0009306.s001.docx]

**S1 Text. Rare structural viral read variants correlate with expression of specific host genes.**

The high coverage corresponding to almost the entire viral genome obtained via viscRNA-Seq enabled further investigation of the structure of viral reads. Among millions of viral reads detected, we observed 14,956 gap reads (~0.1% of total viral reads), defined by having a deletion within read 1 or read 2 (we used Illumina paired-end sequencing, see Methods), not including reads with gaps between the two reads (**S3A Fig**). These gap reads were present in 271 cells and their abundance strongly correlated with vRNA abundance in the same cells, indicating that deep viral coverage was required for detection (**S3B Fig**). The length of these gaps ranged from 20 to over 10,000 nucleotides, with the majority being shorter than 1000 nucleotides (**S3C Fig**). The most common was a 36-base gap located within the coding region of the 6K protein (black arrow in Fig S3A). This gap was found in a total of 1,226 reads derived from 55 different cells. Prediction of the RNA structure via RNAfold web server [(1)](https://paperpile.com/c/HVbaFC/tc6N) revealed that in the presence of the 36-base gap, there is formation of a hairpin with a free energy of -21.23 kcal/mol, indicating a very stable structure (**S3D Fig**). Although the biological function of this hairpin is unknown, stable RNA structures play essential roles in viral replication and tropism across multiple viruses. Alternatively, we cannot currently exclude that this gap could be a result of polymerase errors during the library preparation.
