# Supplementary figures and images for: The transcriptional landscape of Venezuelan equine encephalitis virus (TC-83) infection
Source: PLoS Negl Trop Dis. 2021 Mar 31;15(3):e0009306. doi: 10.1371/journal.pntd.0009306 (PMC8041203; doi:10.1371/journal.pntd.0009306)

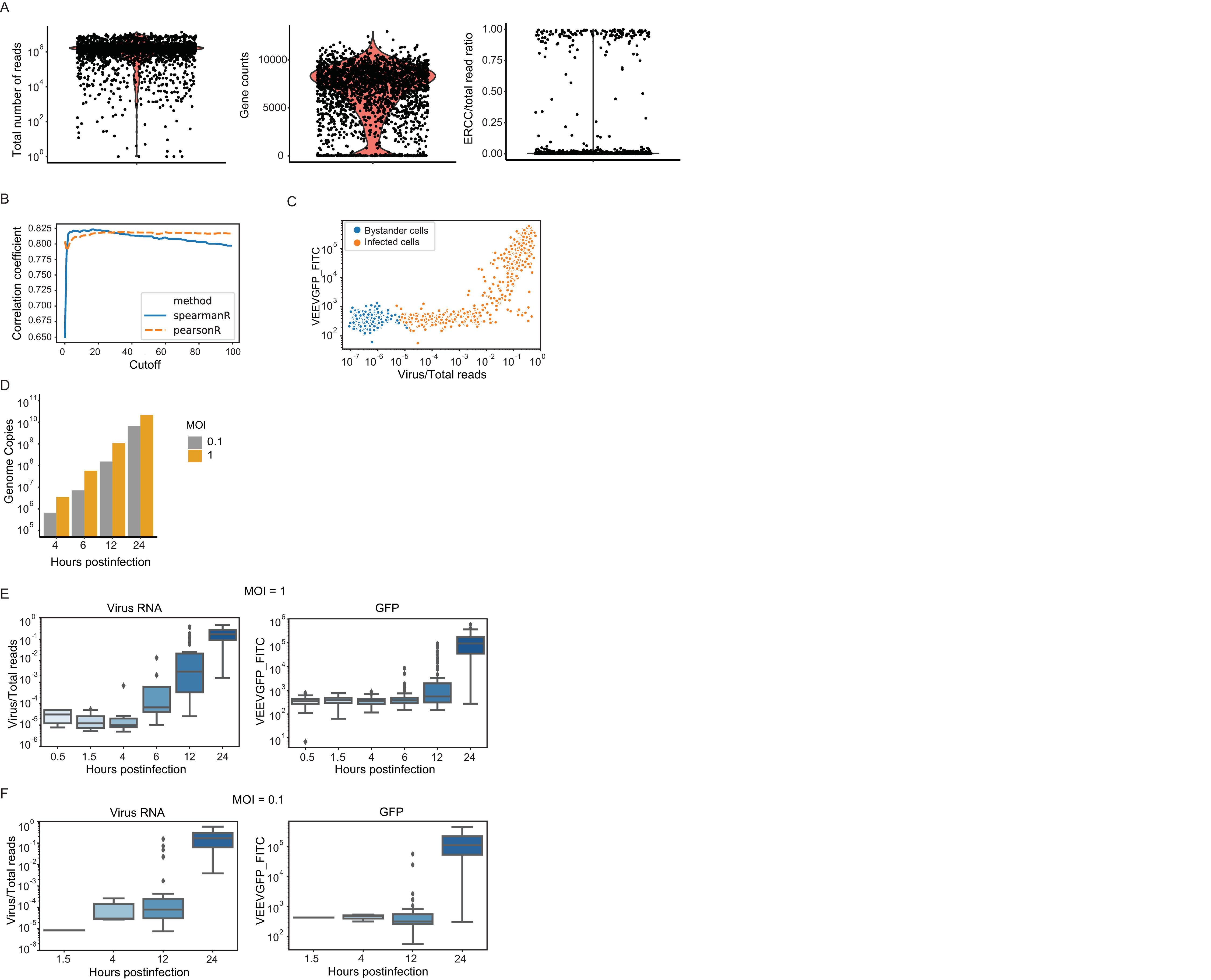

Supplement: S1 Fig — (A) Quality control of the VEEV-TC-83 infected cells dataset. Shown are the total number of reads (left panel), gene counts (middle panel) and ratio of ERCC spike-in RNA reads to total reads (right panel). Cell selection criteria included: total reads > 300,000, gene counts > 500 and a ratio of ERCC spike-in RNA to total reads < 0.05. (B) Spearman’s and Pearson correlation coefficients between GFP expression and vRNA reads when using different cutoffs (from 0 to 100) to define infected cells. (C) Scatter plots showing GFP expression level and vRNA/total reads in cells with detectable vRNA reads. Cells harboring more than 10 vRNA reads. Orange, infected cells; blue, bystander cells. (D) Genome copies of VEEV-TC-83 per 500 ng total RNA at various time points postinfection at MOI 0.1 or 1. (E and F) Box plots showing virus/total read ratio (left panel) and GFP expression level (right panel) over time in infected cells (viral reads > 10) at an MOI of 1 (E) or 0.1 (F) (no infected cells were detected at an MOI of 0.1 at 0.5 and 6 hpi). HPI, hours postinfection; MOI, multiplicity of infection; ERCC, External RNA Controls Consortium. (TIF) [file pntd.0009306.s002.tif]

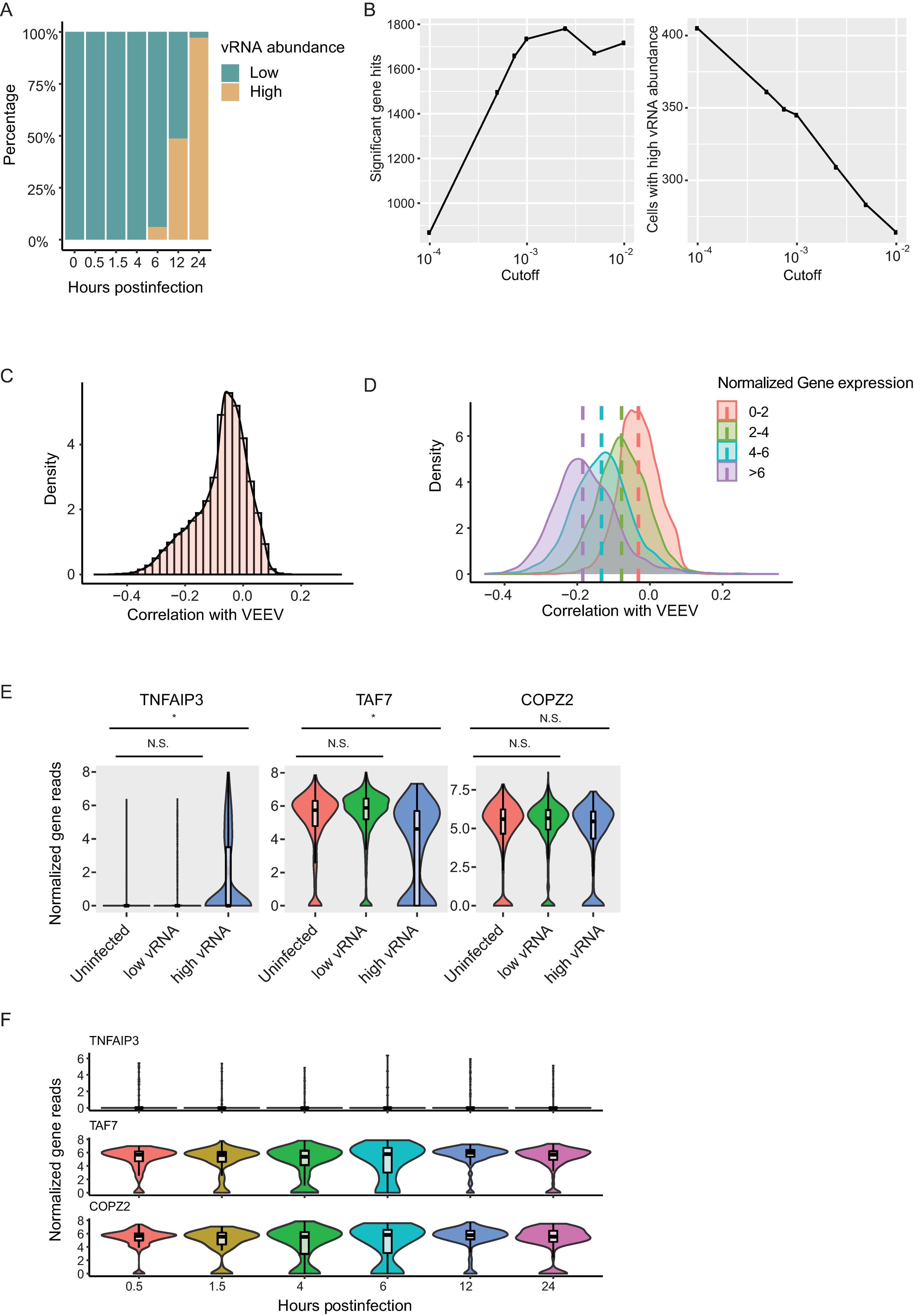

Supplement: S2 Fig — (A) Percentage of low and high vRNA-harboring cells at each time point. High vRNA-harboring cells are defined as virus cDNA reads/total cDNA reads > 0.001. (B) The number of differentially expressed genes (left panel) and cells with high vRNA abundance (right panel) under different cutoffs set by a range of virus cDNA/total reads (from 0.0001 to 0.01). (C) Distribution of Spearman’s correlation coefficients between VEEV-TC-83 vRNA abundance and ~55,000 host genes. (D) Distributions of Spearman’s correlation coefficients shown in D stratified by the average expression level of the gene in uninfected cells. N.S., not significant; (E) Representative genes with distinct expression patterns between uninfected, low vRNA and high vRNA cell groups. *, p < 0.05 by Mann–Whitney U test. (F) The expression of genes shown in (E) does not significantly change over time in uninfected cells. (TIF) [file pntd.0009306.s003.tif]

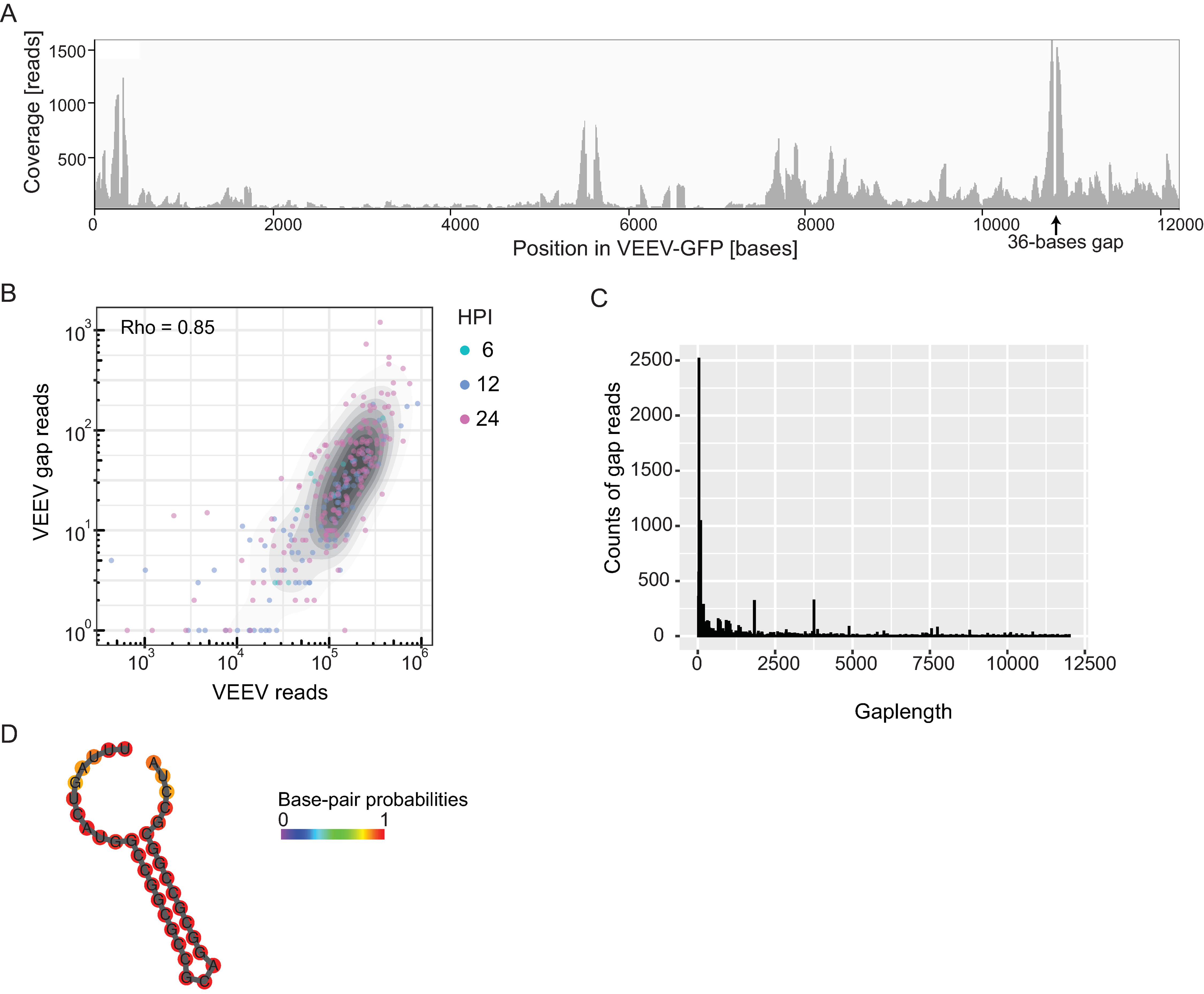

Supplement: S3 Fig — (A) Coverage of VEEV gap reads over the VEEV-TC-83-GFP genome. (B) Scatter plot of number of VEEV gap reads and VEEV total reads within cells with detected gap reads. Each dot represents a cell and colored by hpi. (C) Histogram of gap lengths indicating that the majority of gaps are shorter than 1000 nucleotides. (D) RNA structural prediction of the most common 36-base gap (arrow in A) via RNAfold web server. Scale bar indicates the possibilities of base pairing. Hpi, hours postinfection. (TIF) [file pntd.0009306.s004.tif]

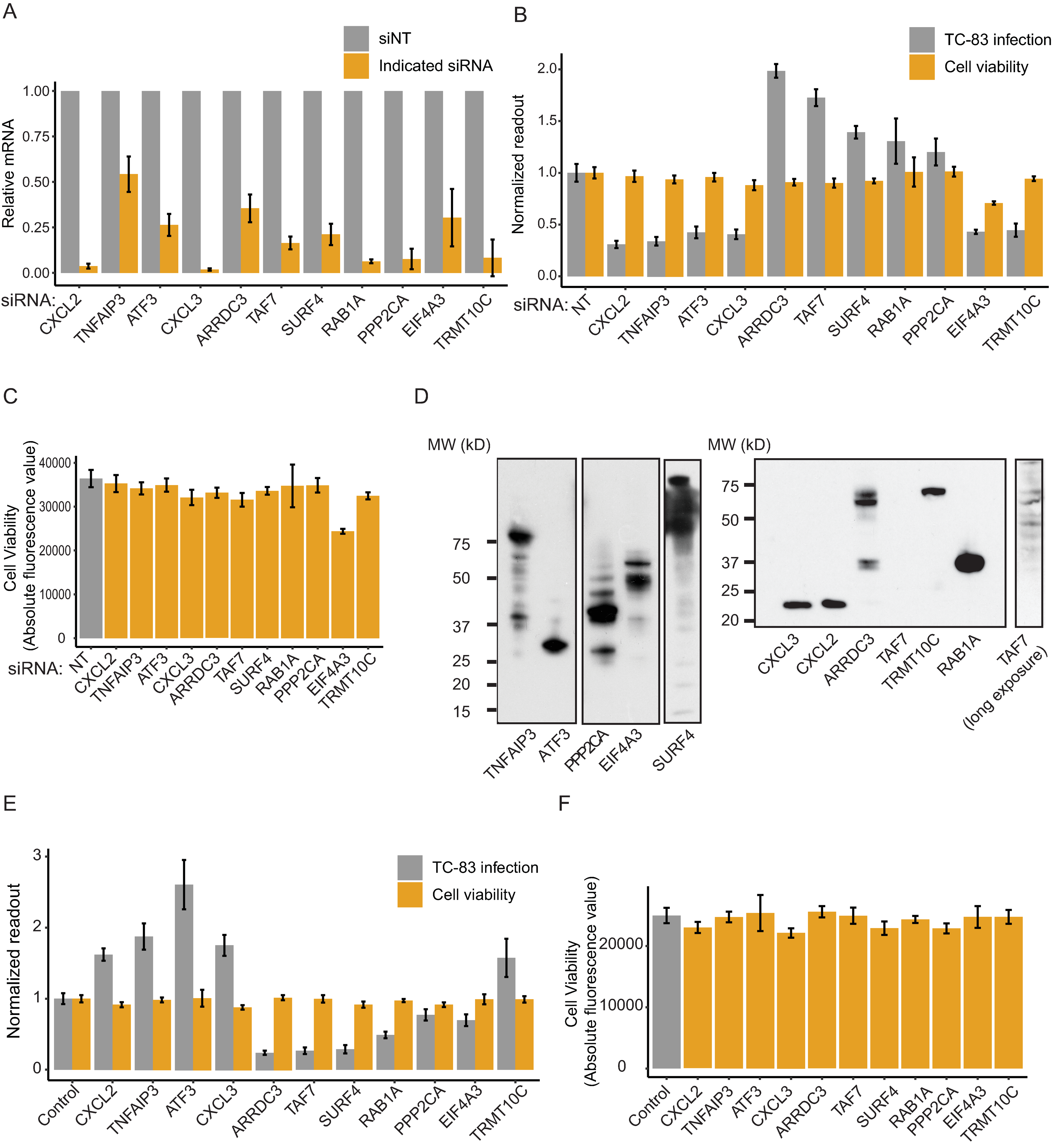

Supplement: S4 Fig — (A) Confirmation of gene expression knockdown in U-87 MG cells transfected with the indicated siRNAs or non-targeting control (NT) via qRT-PCR at 96 hours post-transfection. Results are relative to the level of the respective genes in the NT control. (B and E) Overall VEEV-TC-83 infection (grey) measured by luminescence assays and cell viability (orange) measured by alamarBlue assays in U-87 MG cells transfected with the indicated siRNAs (B) or ectopically expressing the indicated cellular factors (E) at 18 hpi with VEEV-TC-83-nLuc (MOI = 0.01). Data are expressed relative to siNT (B) or empty plasmid (E) controls. (C and F) Absolute fluorescence values from the alamarBlue assays shown in B and E. (D) Confirmation of ectopic expression of the indicated gene products tagged with a FLAG-tag by Western blot in U-87 MG cells. Membranes were blotted with anti-FLAG antibody. Samples in the left panels were run on the same gel from which several lanes were cut out. TAF7 expression on the right is shown at a higher exposure. Data sets are pooled from two independent experiments with six replicates each (B,C, E and F). Shown are means ± SD. (TIF) [file pntd.0009306.s005.tif]

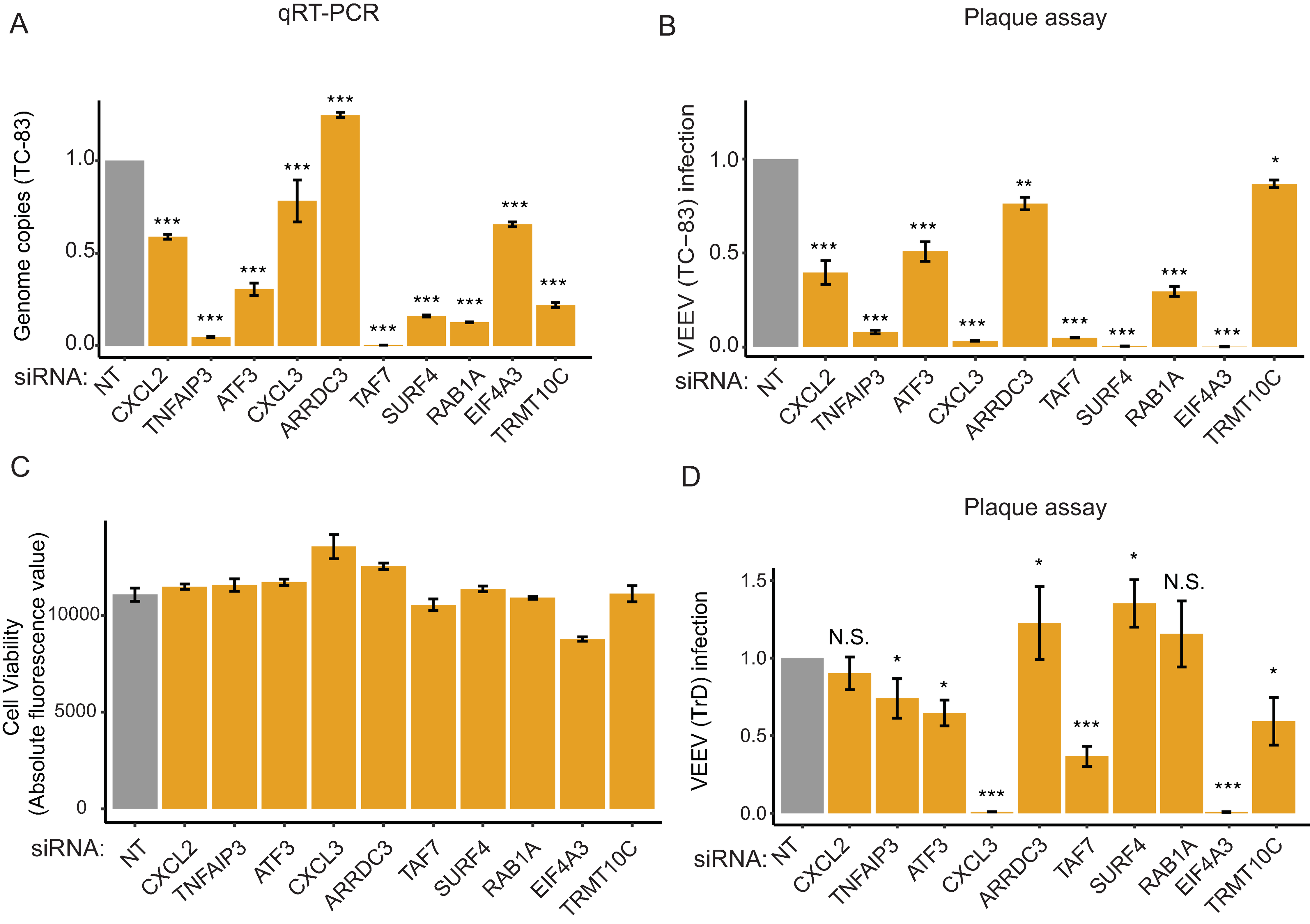

Supplement: S5 Fig — (A) Viral genome copies in lysates derived from U-87 MG cells transfected with the indicated siRNAs 24 hpi with non-reporter VEEV-TC-83 at an MOI of 0.01. (B and D) VEEV infection via plaque assays in U-87 MG cells transfected with the indicated siRNAs 24 hpi with non-reporter TC-83 (B) and TrD (D) (MOI = 0.001). C. Cell viability via alamarBlue assays in U-87 MG cells transfected with the indicated siRNAs. Shown are means ± SD. Data are plotted relative to non-targeting (NT) siRNA control (A, B and D). Representative experiments of at least two conducted are shown. *, q < 0.05; **, q < 0.01; *** q < 0.001 by 1-way ANOVA followed by False Discovery Rate (FDR) corrected multiple comparisons test. N.S, non-significant. (TIF) [file pntd.0009306.s006.tif]

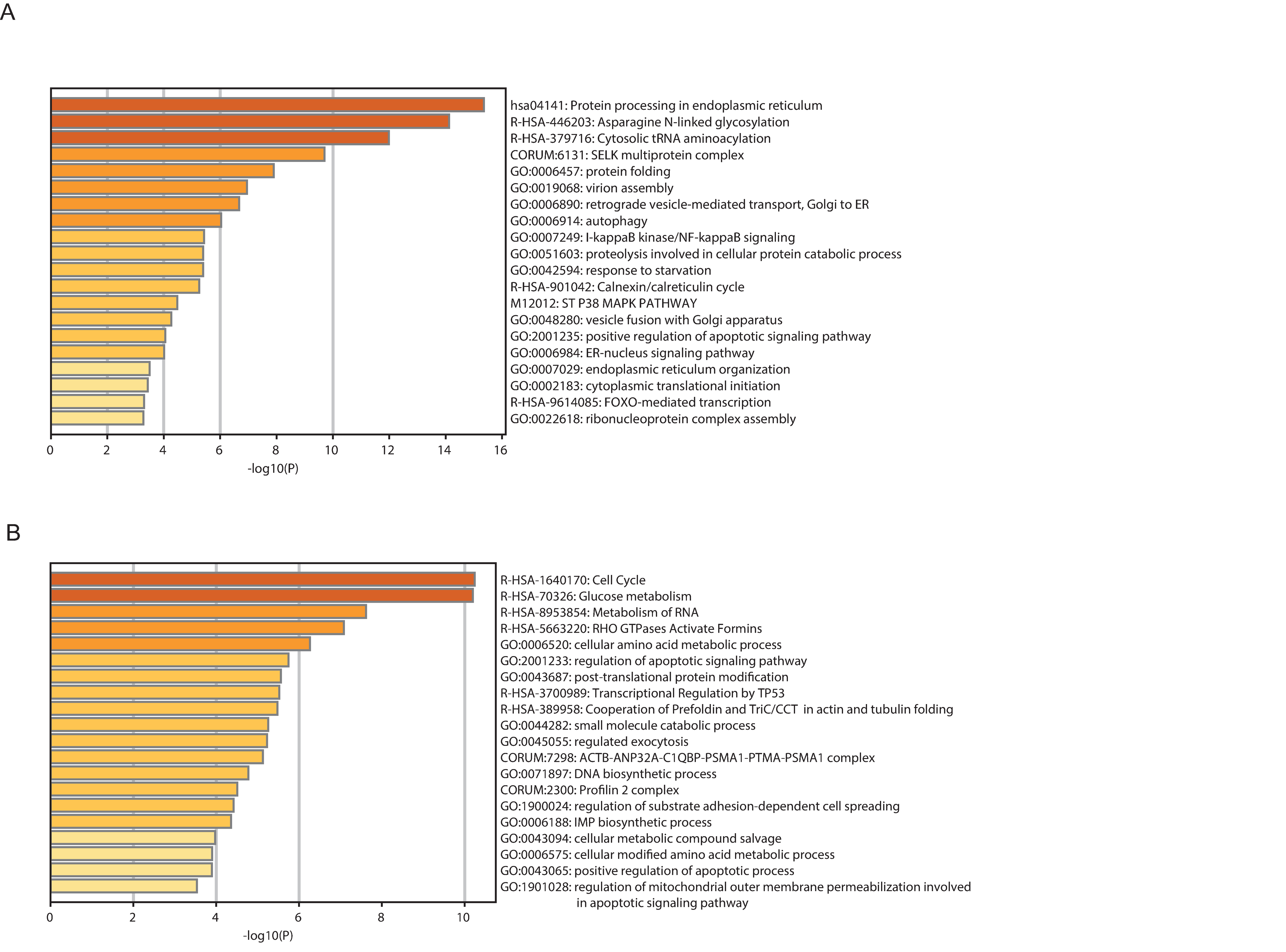

Supplement: S6 Fig — Pathway analysis for genes that positively correlated with VEEV 3’/5’ read ratio and positively (A) or negatively (B) correlated with DENV and ZIKV. Each bar represents a group of genes according to Gene Ontology, KEGG, or other databases of biological function. The plot was made using metascape. (TIF) [file pntd.0009306.s007.tif]
