# Supplementary material for: The transcriptional landscape of Venezuelan equine encephalitis virus (TC-83) infection
Source: PLoS Negl Trop Dis. 2021 Mar 31;15(3):e0009306. doi: 10.1371/journal.pntd.0009306 (PMC8041203; doi:10.1371/journal.pntd.0009306)
Supplement: Attachment — Submitted filename: Response to the reviewers.pdf [file pntd.0009306.s011.pdf]

Response to Reviewers (PNTD-D-20-01090)

Dear Editor,

We thank the Reviewers for being supportive of this manuscript and for their thoughtful and helpful comments. Below we provide a detailed response to each of the items mentioned by the Reviewers and indicate how we have incorporated these suggestions into our revised manuscript. Please, note that all page locations and figure numbers referred to below are with respect to the revised manuscript.

Reviewer #1:

1. The conclusions in some cases appear to be too broad, beyond what is demonstrated by the provided datasets. The limitations of the methodology have not been well described. The rationale for choosing flaviviruses for comparison with alphaviruses has not been described and the relevance to pathology related to alphaviruses is unclear.

--We have now narrowed down our conclusions to reflect that the vaccine VEEV-TC-83 strain was used and that validated hits are potential proviral or antiviral factors. Please, see examples in lines 35 and 45 on Page 2, lines 155-158 on Page 7, and lines 548-549 on page 23.

-Additionally, we have added a paragraph in the Discussion outlining the limitations of the methodology used. Please, see page 32, lines 784-818.

-- The goal of Fig. 6, where flaviviruses are included, is to provide a proof of concept for the utility of the single cell transcriptomic approach in revealing both common and unique signatures via comparison of the host response between multiple unrelated viruses. The rationale for choosing flaviviruses is that they are unrelated to VEEV and, to the best of our knowledge, they are the only other viruses beyond TC-83 to have been studied by the viscRNA-seq platform. This has now been clarified in the text. See page 25, lines 612-614.

2. The authors have used U87MG cells and infected them with TC-83. This is a well established model and there are several manuscripts that are published about transcriptomic changes, although not at the single cell level. While the authors allude to host factors that are published in the literature as being relevant to VEEV multiplication, they do not comment on whether these requirements are indeed at the level of the host transcript.

--In response to the Reviewer’s comment, we have now provided more details regarding the host factors previously reported to affect VEEV infection and stated whether the evidence was at the transcript or protein level. Please see lines 124-132 on page 6.

3. Figure 1 would benefit from an experimental demonstration such as a PCR (a figure) that verifies the infectivity state prior to sample processing (Figure 1A, between the incubation and sample processing steps).

--This has now been done. To verify the infectivity state in the samples prior to processing, we measured viral transcripts in TC-83 infected U87 MG cells at the various time points via qRT-PCR. Please see **Fig. S1D** and lines 399-400 on page 17.

4. In Figure 1C, the increase in the fraction of infected cells between the 6 and the 12 hour time points is too dramatic without a stepwise increase. It may be beneficial to include an additional time point between these two points.

--We agree with the Reviewer that it may be beneficial to add an additional time point, but this would require repeating the entire experiment to avoid batch effects, which is very laborious and expensive. The reviewer's observation also fits well with the sigmoidal dynamics of infection in a cell population: between 6-12 hours from infection to viral RNA production, the number of cells containing vRNA increases much more rapidly than either before or after. Indeed, this nonlinearity of infection presents a challenge in the experimental design, as we have experienced in our previous work on flaviviruses as well. Regardless, however, we feel that this will unlikely change the take home messages of this manuscript.

5. It is not clear what the authors refer to as mock-infected. Ideally, it should be some kind of a replication incompetent virus (UV-inactivated) and comparisons should be made in that context.

--We apologize for this error. The control we used was uninfected cells grown in parallel and subject to the same procedures, except for infection. We have substituted 'mock-infected' with 'uninfected' throughout the manuscript's text and figures.

6. When the authors identify certain pro and antiviral transcripts and attempt to do loss and gain of function assays, no data is included that shows the extent of depletion or overexpression. This is an important piece of information which will be critical to the determination of how much a given transcript actually matters to the virus replication.

--We thank the Reviewer for this important comment, which has now been addressed. We have now included measurements of the transcript level of the depleted factors via qRT-PCR (see **Fig. S4A** and lines 543-544 on page 23).

Additionally, we have included Western Blot analysis showing the level of ectopic expression of the various gene products (see **Fig. S4D** and line 558 on page 23).

7. Absolute cell survival numbers without normalization should be shown for the depletions and the over expression contexts.

--Per the Reviewer's request, we have now included the absolute readout of cell survival. Please, see **Figs. S4C, S4F and S5C**.

8. The impact on the virus is based only on luciferase reporter viruses. The validation should be done in the context of TC-83 (without the reporter) and absolute genomic copy numbers and infectious titre counts should be obtained. More importantly, the relevance to disease cannot be ascertained unless the relevance of these transcripts are measured in the context of disease-causing virus (wild type VEEV).

--This is an important comment that has now been addressed. Per the Reviewer's request, we have conducted loss-of-function experiments in U87-MG cells infected with the wild type non-reporter TC-83 and virulent-TrD VEEV.

First, we performed qRT-PCR to measure viral RNA and plaque assays to measure the infectious titers in cells depleted for the individual cellular factors and infected with non-reporter TC-83. As shown in Figs. S5A-C, while our results are largely consistent with the luciferase assays, some differences were observed. Particularly, TAF7, SURF4 and RAB1A show a proviral phenotype in cells infected with the non-reporter virus vs. an antiviral phenotype in the luciferase assay. We have described our findings and provided potential explanation for this discrepancy in the revised manuscript. See **Figs. S5A-C**, lines 568-585 on page 24, and lines 599-602 on page 24.

Additionally, to probe the relevance of these findings in virulent VEEV infection, we measured the effect of depletion of these cellular factors via siRNAs on VEEV-TrD infection via plaque assays. Depletion of CXCL3 and EIF4A3 dramatically reduced VEEV-TrD infection, similarly to the effect on TC-83 viral titer. Depletion of TNFAIP3, ATF3, TAF7, and TRMT10C mildly to moderately reduced VEEV-TrD infection. ARRDC3 and SURF4 exhibited a phenotype suggestive of antiviral factors in VEEV-TrD, as their depletion increased viral titer. In contrast, depletion of CXCL2 and RAB1A, which suppressed TC-83 viral titers, had no apparent effect on TrD infection. These findings indicate that while some factors are not functionally relevant to virulent VEEV-TrD infection, other factors do appear to be functionally relevant to infection with both viral strains. See **Fig. S5D** and lines 587-602 on pages 24-25.

These findings are also discussed in the Discussion section in lines 720-742 on page 30 and lines 810-818 on page 33.

<u>Reviewer #2:</u>

1. Performing transcriptional profile of a virus infection in cell line derived from malignant glioma is inappropriate as the immortalized cells are by definition not normal and important pathways such as antiviral response may be altered or absent.

--We agree with the Reviewer that immortalized tumor cells, including those derived from malignant glioma, often have defects in the type I IFN pathway. Nevertheless, U87-MG is among the most commonly used cell lines for the investigation of VEEV-host interaction (here are a few examples of many: L Lundberg, et al. Front Microbiol 2018; Baer A, et al. J Virol 2016) Similarly, Huh7 cells have impaired TLR3 pathway and Huh7.5 are defective in both RIG-I and TLR3 pathways, yet, these are commonly used models to study flaviviral (and other viral)-host interactions. Thus, while we agree that this model is imperfect to study some aspects of the host response, it is still a viable model for studying the global host response to VEEV-TC-83 infection.

From the study design perspective, the choice of a permissive cell line was deliberate, because it enabled observation of subtle expression changes that would be much harder to detect in interferon-competent cells. The latter cells typically show a substantial upregulation of hundreds of ISGs and related genes, masking important pathways associated with viral replication but not innate immunity. Indeed, when we first compared our results on dengue virus from IFN-deficient Huh7 cells (Zanini et al. eLife 2018) with primary immune cells (Zanini et al PNAS 2018), this was exactly the dominant difference. Of course, choosing a permissible cell line, while presenting this key advantage, is less informative as of the dynamics of infection in vivo. Yet, the latter is a distinct research direction.

--In response to the Reviewer's comment, we have included paragraphs in the Discussion section outlining the limitations of the U87-MG cell line. Please see lines 795-809 on page 33.

2. The VEE complex is comprised of 6 subtypes and 7 varieties. VEEV-IAB and IC are epidemic viruses, whereas all others are endemic viruses. The utilization of a vaccine strain that by definition is not a wild-type virus is inappropriate as the transcription profile may be vastly different than wild-type viruses.

--We agree with the Reviewer that there may be differences in the cellular transcriptomic response between TC-83 and some or all of the wild type VEE viruses. Nevertheless, while the vaccine strain is not a wild-type virus, it was developed by serial passaging of the virulent, subtype IAB Trinidad donkey (TrD) VEEV strain. Although indeed attenuated, published data indicate that TC-83 maintains some degree of virulence. For example, nearly 40% of vaccinated people develop a systemic febrile illness and other adverse effects (Paessler S, et al. J Virol 2003; Alevizatos AC, et al. Am J Trop Med Hyg 1967). When used to immunize horses, TC-83 can lead to a febrile illness with viremia levels sufficient to infect mosquitoes and initiate a transmission cycle (Henderson BE, et al. Biomed Res Int. 2016). Moreover, in mouse models, TC-83 was shown to be uniformly lethal after intracerebral inoculation and to produce clinical illness for ~14 days after subcutaneous inoculation (Ludwig GV, et al. Am J Trop Med Hyg. 2001).

Moreover, we have conducted loss-of-function experiments in cells infected with wild type VEEV-TrD. Our results suggest that some of the hits are functionally relevant in virulent VEEV infection, supporting that while some aspects of the transcriptome responses between the two strains vary, others are conserved. Please, see response to **comment #8 of Reviewer #1**.

Finally, this study establishes the feasibility and utility of applying viscRNA-Seq to VEE viruses and opens the door for future studies on wild-type strains, which are much more challenging in practice because they require higher biosafety containment facilities. Indeed, the comparison between TC-83, its parental strain, and other subtypes within the VEE complex or other alphaviruses is a very interesting suggestion for future studies connecting viral evolution with viral-host interactions.

In response to the Reviewer's comment, we have described this limitation of our study in the Discussion. Please see lines 784-795 on page 32 and lines 810-818 on page 33.

3. TC-83 attenuation is due to 2 point mutations; 5' UTR nucleotide G3A and E2-120 Thr-to-Lys. The 5' UTR point mutation is in a structural element that renders the vaccine sensitive to inhibition by Ifit1, whereas the wild-type virus resistant to inhibition by Ifit1. This host-pathogen interaction suggests that there may be considerable differences between the TC-83 and VEEV-IAB in transcriptional profiles.

--We have now described the mutations in the TC-83 strain and stated the limitation in studying some aspects of the host response. Please see page 32-33, lines 784-795.

4. The second attenuating point mutation in E2 of TC-83 virus is an adaptation to Heparan sulfate receptor. This adaptation enables rapid adsorption of virus in susceptible cell lines. This rapid adsorption in cells producing infectious virus particles will lead to re-adsorption of virus particles upon release. This effect may explain the "superproducer" effect and the subsequent transcriptional profile difference.

--Indeed, as pointed out by the Reviewer, the E2 point mutation of TC-83 is an adaptation to use heparan sulfate as an attachment receptor and it reduces lethality of the virus in mice. Nevertheless, this mutation is presumably present in all viral particles in the viral stock used in our study. All the cells were infected by the same mutation-harboring viral stock and processed together, yet only a small subset of these cells show the superproducer effect at 6 hours postinfection. It is therefore unlikely that this mutation explains the "superproducer effect".

Our experimental design did not include a specific mechanism to inhibit or distinguish self-infection by virions that were just released from cells. Such studies on influenza virus (Russel et al. eLife 2018), however, have indicated that although the fraction of infected cells and the average amount of vRNA within cells decrease when reinfection is eliminated, the biological conclusions of the scRNA-Seq are very similar.

As mentioned in the answer to question #2 by the same Reviewer, a promising future direction of the current research is comparing at the single cell level the current TC-83 experiment with another experiment involving virulent strains. The connection between "superproducers" and heparan sulfate binding and other questions could be then addressed directly. However, those

experiments require significant funding and commitment since they are performed in BSL3-level biosafety facilities.

Reviewer #3:

1. The work described in this study was performed with a live-attenuated vaccine strain – TC-83 because it also replicates rapidly. The discussion would benefit from a sentence or 2 on why this strain is representative of what we would expect with a fully virulent strain, and that the host genes identified are likely to be the same. This is meant for a more general audience who is not familiar with VEEV and its vaccine strain.

-- We have now included this information. Please, see lines 83-87 on page 4 and lines 784-795 on page 32.

2. One aspect I would have liked the authors to maybe elaborate a bit more on, probably in the discussion, is the observation of gapped reads. These could indicate the presence of defective virus genomes, which are observed in many RNA viruses. There are a number of recent studies on defective viruses in other systems, including scRNAseq studies of influenza, and the potential of these defective genomes in interference. The authors indicate that they can't determine if these gapped reads are an artifact of the polymerase during amplification but they could mention something about defective virus genomes not being an uncommon phenomenon. The authors do make a point to provide text and data in the supporting information on what they observe, so this is a minor point.

--This is an insightful comment that has now been addressed. We have added that it is possible that our finding represent DVGs and included several relevant references. Please see lines 535-537 on page 22.

3. The text in parenthesis should be reversed to show "(virus reads/total reads > 0.001)" and "(virus reads/total reads < 0.001)". The way it is currently stated is confusing.

--In response to Reviewer's comment, we have now changed these titles.

4. Line 391: The text in parenthesis should be reversed to show "(virus reads/total reads > 0.001)" and "(virus reads/total reads < 0.001)". The way it is currently stated is confusing.

--This has now been changed.

5. Fig 2A: there is a typo in the label pointing to the green circle -- "differntial"

--This error has now been fixed.

6. Fig S1: Panel E GFP panel needs a label on the Y axis.

--Thanks for pointing this error out. This has now been fixed.
